# Partnerschaftliche Gewalt während der COVID-19-Pandemie in der Schweiz

**DOI:** 10.1007/s11757-021-00698-1

**Published:** 2022-01-10

**Authors:** Dirk Baier, Lorenz Biberstein, Sören Kliem

**Affiliations:** 1 Institut für Delinquenz und Kriminalprävention, Departement Soziale Arbeit, Zürcher Hochschule für Angewandte Wissenschaften, Zürich, Schweiz; 2 Fachbereich Sozialwesen, Ernst-Abbe-Hochschule Jena, Jena, Deutschland

**Keywords:** Häusliche Gewalt, Befragung, Partnerschaft, Trendanalyse, Drohungen, Domestic violence, Survey, Partnership, Trend analysis, Threats

## Abstract

Der Beitrag berichtet Ergebnisse zu 2 schweizweit repräsentativen Befragungsstudien. Im Jahr 2018 und erneut im Jahr 2021 wurden Erwachsene, die zum Befragungszeitpunkt mit einem Partner bzw. einer Partnerin zusammenlebten, nach dem Erleben verschiedener partnerschaftlicher Übergriffe in den letzten 12 Monaten gefragt. Die Ergebnisse zeigen, dass es im Zeitvergleich nicht zu einem Anstieg partnerschaftlicher Gewalt gekommen ist. Der Anteil an Befragten, die psychische Gewalt erlebt haben, beträgt 13,8 (Befragung 2018) bzw. 11,7 % (Befragung 2021), der Anteil an Befragten, die physische Gewalt erlebt haben, 2,9 bzw. 3,1 %. Die Stabilität der Prävalenzraten zeigt sich allerdings nicht für alle demografischen Gruppen: Bei jüngeren Befragten findet sich ein Anstieg physischer Gewalterfahrungen.

## Abstract (en)

The article reports results from two nationally representative survey studies from Switzerland. In 2018 and again in 2021 adults who lived together with a partner at the time of the survey were asked about their experience of various forms of partner violence in the last 12 months. The results show that there has not been an increase in intimate partner violence over time. The proportion of respondents who experienced psychological violence was 13.8% (2018 survey) and 11.7% (2021 survey), while the proportions of respondents who experienced physical violence were 2.9% and 3.1%, respectively; however, a stability of prevalence rates was not found for all demographic groups: Among younger respondents, there was an increase in experiences of physical violence.

## Einleitung

Laut *Polizeilicher Kriminalstatistik* (Bundesamt für Statistik 2020a) wurden im Jahr 2019 schweizweit 19.669 Delikte häuslicher Gewalt registriert. Bei der Hälfte der Delikte (48,6 %) handelte es sich um Gewalt zwischen Personen in einer Paarbeziehung, bei einem Viertel handelte es sich (25,5 %) um Gewalt zwischen ehemaligen Partnerinnen und Partnern – partnerschaftliche Gewalt macht insofern einen Großteil der registrierten häuslichen Gewalt aus. Mit dem Beginn der COVID-19-Pandemie und der Einführung von Maßnahmen zum Infektionsschutz der Bevölkerung und zur Eindämmung der Pandemie, die u. a. Ausgangsbeschränkungen und Homeoffice- sowie Homeschooling-Regelungen beinhalteten, wurde die Sorge geäußert, dass Gewalt im häuslichen Bereich steigen könnte. Bislang liegen weitestgehend nur Daten der *Polizeilichen Kriminalstatistik*, des sog. Hellfelds, dazu vor, inwieweit sich diese Sorge bestätigt hat.

Die Anzahl an registrierten Delikten häuslicher Gewalt lag im Jahr 2020 mit 20.123 2,3 % über der Anzahl von 2019 (Bundesamt für Statistik 2021a); dies deutet zunächst auf einen Pandemieeffekt entsprechend den Erwartungen zur Entwicklung der häuslichen Gewalt hin. Auch die vom Bundesamt für Statistik (2021b) durchgeführte Sonderauswertung konstatiert: „Im Jahr 2020 wurden im Vergleich zum Mittelwert der letzten drei Jahre 5 % mehr Straftaten (+155) im häuslichen Bereich polizeilich registriert, die während der außerordentlichen Lage (16.03.2020 bis 19.06.2020 […]) stattfanden.“ Allerdings ist in Bezug auf diese Anstiege zu beachten, dass die Anzahl registrierter Straftaten häuslicher Gewalt seit 2011 – mit Unterbrechung in den Jahren 2014 und 2017 – kontinuierlich steigt, durchschnittlich jährlich um 3,7 % (2012–2019); bereinigt um die Bevölkerungsentwicklung (ansteigende Bevölkerungszahlen in der Schweiz) ergibt sich ein durchschnittlicher jährlicher Anstieg von 2,6 % (2012–2019). Dies bedeutet, dass auch abseits von Pandemieeffekten die Raten von häuslicher Gewalt von Jahr zu Jahr zugenommen haben, womöglich auch deshalb, weil sich die gesellschaftliche Sensibilität und das hiermit verbundene informelle Kontrollverhalten erhöht haben und zunehmend Anzeige erstattet wird. Denkbar ist also auch, dass im Vergleich von 2019 und 2020 auch ohne Pandemie ein Anstieg der Zahlen zu erwarten gewesen wäre, der dann sogar mit 2,3 % hinter dem zugrunde liegenden zeitlichen Trend von 3,7 % zurückbleibt. Ein Pandemieeffekt lässt sich mit den Daten der *Polizeilichen Kriminalstatistik* demnach nicht nachweisen.

Die Daten der *Polizeilichen Kriminalstatistik* sind hinsichtlich ihrer Aussagekraft freilich deshalb begrenzt, weil sie maßgeblich von der Anzeigebereitschaft abhängen. Wie Biberstein und Killias (2016, S. 20 f.) anhand einer schweizweiten Befragung feststellen, wird maximal ein Drittel der Gewaltvorfälle im häuslichen Bereich (untersucht wurden sexuelle Gewalt und Tätlichkeiten/Drohungen) bei der Polizei angezeigt. Studien aus Deutschland weisen auf eine noch niedrigere Anzeigerate hin. Hellmann (2014, S. 123) beispielsweise folgert auf Basis einer umfassenden Befragung: „Von allen Fällen häuslicher Gewalt durch die Partnerin bzw. den Partner gelangten der Polizei und Staatsanwaltschaft 14,7 % … zur Kenntnis“. Eine Annahme ist, dass die Pandemiesituation die Anzeigebereitschaft noch einmal gesenkt hat, u. a. deshalb, weil die Täter*innen die Opfer noch engmaschiger kontrollieren und damit verhindern können, dass diese sie anzeigen. Zudem können Schulen, Vereine und andere Organisationen ihre Rolle bezüglich der Aufdeckung häuslicher Gewalt schlechter ausüben, wenn sich mögliche Opfer dort seltener aufhalten, was aufgrund von Schulschließungen und des temporären Aussetzens von Vereins- und anderen Freizeitaktivitäten de facto der Fall war. Auch wenn die Effekte der Pandemiesituation auf das Anzeigeverhalten möglicherweise überschätzt werden – so geben Opfer beispielsweise nur selten an, dass sie keine Anzeige erstatten, weil sie vom Täter bedroht werden (Hellmann 2014, S. 128), und Anzeige wird in den allermeisten Fällen von den Opfern, seltener noch von Freund*innen erstattet und kaum von Organisationen im Umfeld (Hellmann 2014, S. 123 f.) –, bleibt grundsätzlich der Nachteil dieser Datenquelle bestehen, dass sie nur einen Teil des Gewaltgeschehens sichtbar machen kann und insofern für die Prüfung eines Pandemieeffekts eher ungeeignet erscheint.

Die bisher vorhandenen Studien zum Zusammenhang von COVID-19-Pandemie und häuslicher Gewalt beschränken sich aber weitestgehend auf diese Datengrundlage. So berichten Piquero et al. (2021) Ergebnisse einer 18 Einzelstudien umfassenden Metastudie, die sich sämtlich auf Hellfelddaten beziehen, wobei dies nicht immer polizeiliche Kriminalstatistiken, sondern auch beispielsweise Krankenhausdaten (Einweisungen in Notaufnahmen) sind. Hauptergebnis ist, dass Ausgangsbeschränkungen/Lockdowns während der Pandemie mit einem 8,1-prozentigen Anstieg der häuslichen Gewalt einhergehen. Gleichwohl finden sich auch Studien auf Basis von Kriminalstatistiken, die eine Stabilität bzw. sogar Rückgänge häuslicher Gewalt für diesen Zeitraum berichten (Gerell et al. 2020; Halford et al. 2020). Auch Piquero et al. (2020) kommen anhand ihrer Analysen für eine US-Stadt zu folgendem Ergebnis: „we do not see, at least with the data we have, any lasting increase or sustained higher levels of domestic violence“.

Zweifellos stellen aber wiederholt durchgeführte Dunkelfeldbefragungen zum Themenfeld der partnerschaftlichen Gewalt einen geeigneteren Weg dar, um den Einfluss der COVID-19-Pandemie zu untersuchen, weil die Anzeigebereitschaft und deren mögliche Entwicklung hier nicht von Bedeutung sind. Noch liegen aber solche Studien kaum vor. Verschiedene Befragungsstudien haben daher einen anderen Weg gewählt, indem sie retrospektiv den Einfluss der Pandemie abzuschätzen versuchten. In Australien wurde im Mai 2020 eine Befragung unter Frauen durchgeführt, in der die letzten 3 Monate vor der Befragung hinsichtlich des Gewalterlebens eingeschätzt werde sollten (Boxall et al. 2020). In diesem Zeitraum erlebten 4,6 % der Frauen physische oder sexuelle Gewalt. Entsprechend den Ergebnissen der Studie hat der Lockdown zu einer Erhöhung häuslicher Gewalt beigetragen: Zwei Drittel (65,4 %) der Frauen, die Gewalterfahrungen in Bezug auf die 3 Monate berichteten, erlebten ihrer Aussage nach „either violence for the first time by that partner or an escalation in the frequency and severity of prior violence“ (S. 12). Eine ebenfalls im Mai 2020 im Kanton Zürich durchgeführte Befragung kommt hingegen zu einem anderen Befund: „Es kann weder von einem deutlichen Anstieg noch von einem deutlichen Rückgang häuslicher Gewalt während des Lockdowns gesprochen werden. Der Anteil an Befragten, die von einem Rückgang der einzelnen Übergriffsformen [partnerschaftlicher Gewalt; d. A.] berichten, entspricht weitestgehend dem Anteil an Befragten, die von einem Anstieg während des Lockdowns berichten“ (Baier 2020, S. 458). Auch in dieser Befragung sollten die Befragten retrospektiv einschätzen, ob es ihrer Ansicht nach während des Lockdowns zur häufigeren Gewaltanwendung durch den Partner bzw. die Partnerin kam oder nicht.

Jenseits von Befragungsstudien findet sich für die Schweiz noch eine Studie, die Anrufe bei der „Dargebotenen Hand“ (der Schweizer Telefonseelsorge) analysiert hat. Diese kommt zu dem Befund, dass während des Lockdowns im Frühjahr 2020 die Anrufe wegen häuslicher Gewalt im Vergleich zum entsprechenden Vorjahrszeitraum um 25 % gesunken sind (Brülhart und Lalive 2020).

Bislang kann in der Literatur nur eine Studie identifiziert werden, die tatsächlich Raten häuslicher Gewalt vor und während der Pandemie auf Basis von 2 unabhängigen Befragungen und damit im Sinne einer Pre- und Postmessung miteinander vergleicht: Kliem et al. (2021) berichten Zwölfmonatsprävalenzraten partnerschaftlicher Gewalt anhand einer Befragung des Jahres 2016 (1.317 Befragte in Partnerschaft) und einer Befragung des Jahres 2021 (1.005 Befragte in Partnerschaft). Die Ergebnisse zeigen hinsichtlich der Viktimisierungserfahrungen für weibliche Befragte eine Zwölfmonatsprävalenz zum Zeitpunkt 2016 von 9,3 % und für männliche Befragte von 9,1 %. Für keine der beiden Gruppen konnte eine statistisch bedeutsame Veränderung der Zwölfmonatsprävalenzraten vom Beobachtungsjahr 2016 zu 2021 festgestellt werden. Eigene Gewalthandlungen innerhalb der zurückliegenden 12 Monate wurden zum Zeitpunkt 2016 von 7,3 % der weiblichen und von 9,3 % der männlichen Befragten berichtet. Auch hier ergaben sich über die Beobachtungsjahre hinweg keine statistisch bedeutsamen Veränderungen. Partnerschaftliche Gewalt ist entsprechend diesen Analysen in der Pandemiezeit damit auf einem vergleichbaren Niveau wie vor der Pandemiezeit.

Die vorliegende Studie berichtet erstmals für die Schweiz auf Basis von 2 Repräsentativbefragungen die Prävalenzraten partnerschaftlicher Gewalt und erlaubt damit auf Basis der Betrachtung des Dunkelfeldes eine Antwort auf die Frage, ob in der Schweiz die COVID-19-Pandemie mit Veränderungen partnerschaftlichen Gewaltverhaltens verbunden ist oder nicht.

## Methode und Stichproben

Die partnerschaftliche Gewalt wurde im Rahmen von 2 schweizweit repräsentativen Erwachsenenbefragungen erfasst, die in den Jahren 2018 und 2021 durchgeführt wurden (Baier 2019). Die Befragung des Jahres 2018 erfolgte als schriftliche, postalische Befragung, die Befragung des Jahres 2021 als Online-Befragung. Um zu einer repräsentativen Stichprobe zu gelangen, wurden verschiedene Wege beschritten: Im Jahr 2018 wurden schweizweit per Zufall Adressen gezogen; dies erfolgte durch ein Marketing-Unternehmen, wobei insgesamt 10.749 Adressen bzw. Personen in die Stichprobe einbezogen wurden. Im Jahr 2021 wurde auf das Panel des Markt- und Sozialforschungsunternehmens LINK zurückgegriffen, wobei 18.686 Einladungen verschickt wurden. An der Befragung des Jahres 2018 beteiligten sich letztlich 2.111 Personen, was einer Rücklaufquote von 20,1 % entspricht, an der Befragung des Jahre 2021 3.010 Personen (Rücklaufquote 16,1 %). Bei beiden Fragen entsprach die soziodemografische Zusammensetzung nicht exakt der Zusammensetzung der Grundgesamtheit, weshalb eine Anpassungsgewichtung an die Alters- und Geschlechtsverteilung der Schweizer Bevölkerung erfolgte. Alle in diesem Beitrag berichteten Ergebnisse basieren auf gewichteten Stichproben.

In die nachfolgenden Auswertungen werden nur Befragte einbezogen, die auf die Frage „Hatten Sie in den letzten 12 Monaten eine*n Lebenspartner*in?“ mit „ja“ antworteten und die ebenfalls angaben, mit der Partnerin bzw. dem Partner gemeinsam in einem Haushalt zu leben. Dies waren in der Befragung 2018 1.503 Personen, in der Befragung 2021 1.916 Personen. Die soziodemografische Zusammensetzung dieser Substichproben ist in Tab. 1 dargestellt.

**Table 1:** 

	Befragung 2018	Befragung 2021	
Anteil weiblich in %	45,4	47,7	χ^2^ = 1,795
Alter in Jahren, Mittelwert (Min–Max)	48,19 (20–85)	49,53 (19–79)	t = −2,710**
Anteil, Migrationshintergrund in %	22,8	16,7	χ^2^ = 20,110***
Region Westschweiz in %	24,3	23,7	χ^2^ = 0,670
Region Tessin in %	3,7	4,2	
Bildung mittel – Sekundarstufe II – in %	40,8	45,2	χ^2^ = 6,920*
Bildung hoch – Tertiärstufe – in %	56,0	51,5	
Ort unter 5000 Einwohner in %	38,5	43,8	χ^2^ = 17,023***
Ort unter 20.000 Einwohner in %	34,6	35,0	
Arbeitslos, Bezug von Arbeitslosengeld/Sozialhilfe	2,3	2,5	χ^2^ = 0,129

*p < 0,05, **p < 0,01, ***p < 0,001

Der Anteil weiblicher Befragter beträgt in der Stichprobe des Jahres 2018 45,4 %, in der Stichprobe des Jahres 2021 47,7 %; der Unterschied wird als nicht signifikant ausgewiesen. Ebenfalls keine signifikanten Unterschiede zeigen sich für die Zusammensetzung der Stichproben nach Region (zu 100 % fehlend: deutschsprachige Schweiz jeweils 72,1 %) und Arbeitslosigkeit bzw. Bezug von Arbeitslosengeld/Sozialhilfe. Demgegenüber unterscheiden sich beide Stichproben signifikant hinsichtlich des Alters, des Anteils an Personen mit Migrationshintergrund, der Bildung (zu 100 % fehlend: niedrige Bildung jeweils 3,3 %) und der Einwohnerzahl der Wohngemeinde (zu 100 % fehlend: ab 20.000 Einwohner 26,9 bzw. 21,2 %). Um den Migrationshintergrund zu bestimmen, wurden das Geburtsland und die Staatsangehörigkeit herangezogen; sobald ein Geburtsland außerhalb der Schweiz oder eine nichtschweizerische Staatsangehörigkeit (ggf. zusätzlich zur Schweizer Staatsangehörigkeit) berichtet wurde, wird von einem Migrationshintergrund ausgegangen. Die Einwohnerzahl des Wohnorts basiert auf der Einschätzung der Befragten. Insofern verschiedene der soziodemografischen Merkmale, zu denen signifikante Unterschiede zwischen den Stichproben bestehen, im Zusammenhang mit partnerschaftlicher Gewalt stehen können, sind sie bei den nachfolgenden Auswertungen zu berücksichtigen.

## Ergebnisse

In beiden Befragungen wurden die Teilnehmenden gebeten anzugeben, ob sie in den letzten 12 Monaten verschiedenen Handlungen vonseiten des Lebenspartners bzw. der Lebenspartnerin ausgesetzt waren. Die Handlungen sind in Tab. 2 aufgeführt; die Antwortkategorien waren „nein“ bzw. „ja“, d. h., es wurde darauf verzichtet, die Häufigkeit der Gewaltausübung zu erfassen. Erhoben wurde ausschließlich die Opferperspektive, wobei sich auf die Handlungen der Lebenspartner*innen untereinander konzentriert wurde, d. h., Gewalthandlungen von bzw. gegen Kinder wurden nicht erfasst.

**Table 2:** 

	Befragung 2018	Befragung 2021	
… Lächerlich gemacht, gedemütigt und seelisch verletzt	8,8	8,1	χ^2^ = 0,443
… Mich kontrolliert (z. B. auf Natel nachgeschaut, mit wem ich Kontakt hatte; Kontakte zu Freunden untersagt; danach gefragt, wo ich gewesen bin)	8,7	5,7	χ^2^ = 11,156***
*Index: psychische Gewalt*	*13,8*	*11,7*	*χ*^*2*^ *=* *3,228*
… Mich weggeschubst, getreten, geohrfeigt, gebissen oder gekratzt, sodass es mir wehtat oder ich Angst bekam	2,1	1,5	χ^2^ = 1,835
… Etwas nach mir geworfen, das mich hätte verletzen können	1,7	1,8	χ^2^ = 0,008
… Mich zu sexuellen Handlungen gezwungen, die ich nicht wollte	0,5	0,4	χ^2^ = 0,049
… Mich verprügelt oder zusammengeschlagen	0,3	0,2	χ^2^ = 0,121
… Mich mit einer Waffe verletzt (z. B. Messer, Flasche, Stock)	0,0	0,2	χ^2^ = 3,132
*Index: physische Gewalt*	*2,9*	*3,1*	*χ*^*2*^ *=* *0,194*
… Mir bedrohende Nachrichten (SMS, Chats, Tweets) geschickt	1,0	0,7	χ^2^ = 0,736
… Mir gedroht, mich körperlich anzugreifen oder zu verletzen	0,7	0,8	χ^2^ = 0,154
… Mich mit einer Waffe bedroht (z. B. Messer, Flasche, Stock)	0,1	0,3	χ^2^ = 0,667
*Index: Bedrohung*	*1,5*	*1,5*	*χ*^*2*^ *=* *0,031*
*Index: Gewalt insgesamt*	*14,8*	*13,0*	*χ*^*2*^ *=* *2,498*

*p < 0,05, **p < 0,01, ***p < 0,001

Die Ergebnisse aus Tab. 2 zeigen erstens, dass psychische Gewaltformen häufiger vorkommen als physische Gewaltformen und Drohungen. Zweitens ergibt sich eine Gesamtgewaltrate, die deutlich unter den Raten liegt, die in verschiedenen in jüngerer Zeit in der Schweiz veröffentlichten Studien zum Themenfeld Gewalt gegen Frauen bzw. häusliche Gewalt berichtet wurden (u. a. Bütikofer et al. 2021; Golder et al. 2019), was u. a. mit methodischen Limitationen dieser Studien (u. a. Baier 2019, S. 34 ff.) und dem Berichten von Lebenszeitprävalenzen zu begründen ist. In der vorliegenden Stichprobe gaben 14,8 bzw. 13,0 % der Befragten an, mindestens eine der aufgeführten Formen partnerschaftlicher Gewalt in den zurückliegenden 12 Monaten erlebt zu haben.

Der Fokus der Auswertungen liegt allerdings auf den Veränderungen im Zeitvergleich, wobei sich drittens zeigt, dass bei nahezu allen Gewaltformen sowie bei allen Gewaltindizes keine signifikanten Veränderungen festzustellen sind. Das Ausmaß partnerschaftlicher Gewalt vor und während der COVID-19-Pandemie ist damit weitestgehend identisch. Die psychische Gewalt ist in nichtsignifikanter Weise von 13,8 auf 11,7 % zurückgegangen; in Bezug auf diesen Gewaltbereich findet sich zugleich die einzige signifikante Veränderung, nach der das Kontrollverhalten von 8,7 auf 5,7 % gesunken ist. Physische Gewalt ist von 2,9 auf 3,1 % nicht signifikant gestiegen. Bei der Bedrohung hat es keine Veränderung geben (jeweils 1,5 %).

Um auszuschließen, dass die Vergleiche der Zwölfmonatsprävalenzen durch soziodemografische Faktoren beeinflusst sind, wurden multivariate Analysen mit den 4 Indizes berechnet. In Tab. 3 sind die Ergebnisse von binär-logistischen Regressionsanalysen dargestellt. Neben den Haupteffekten der soziodemografischen Variablen wurden zusätzlich Interaktionseffekte dieser mit dem Erhebungsjahr in den Modellen berücksichtigt; die Interaktionseffekte prüfen, ob es evtl. einen Einfluss des Erhebungsjahres für soziodemografische Untergruppen gibt, d. h., ob sich für diese Untergruppen die Raten in unterschiedlicher Weise entwickelt haben. Das zentrale Ergebnis der Analysen ist, dass zum Erhebungsjahr in keinem Modell ein signifikanter Einfluss festzustellen ist. Im Vergleich der Jahre hat es also weder einen signifikanten Anstieg noch einen signifikanten Rückgang der partnerschaftlichen Gewalt gegeben. Die Pandemiesituation hat sich insofern nicht auf die Gewalt ausgewirkt. Hinzuweisen ist an dieser Stelle darauf, dass alle Koeffizienten zum Erhebungsjahr unter 1 liegen, d. h., dass das Gewaltniveau im Jahr 2021 (nicht signifikant) niedriger ausfällt als 2018.

**Table 3:** 

	Modell: psychische Gewalt	Modell: physische Gewalt	Modell: Bedrohung	Modell: Gewalt insgesamt
Jahr 2021 (Referenz: 2018)	0,870	0,845	0,878	0,878
Geschlecht: weiblich (Referenz: männlich)	0,889	0,969	0,661	0,960
Alter in Jahren	0,980***	0,951***	0,973*	0,976***
Migrationshintergrund	0,916	1,010	1,079	0,882
Region Westschweiz/Tessin (Referenz: deutschsprachige Schweiz)	0,745*	0,952	1,308	0,780*
Bildung hoch (Referenz: Bildung mittel/niedrig)	1,103	0,898	0,803	1,088
Ort unter 5000 Einwohner (Referenz: Ort ab 5000 Einwohner)	1,402**	0,672	0,716	1,364**
Arbeitslos, Bezug von Arbeitslosengeld/Sozialhilfe	2,143**	1,199	2,253	2,033*
Interaktion Jahr * Geschlecht	0,659	0,664	0,494	0,645*
Interaktion Jahr * Alter	1,002	0,962*	0,957*	0,997
Interaktion Jahr * Migrationshintergrund	1,205	2,477	1,655	1,424
Interaktion Jahr * Region Westschweiz/Tessin	0,754	0,964	0,334	0,676
Interaktion Jahr * Bildung hoch	0,599*	1,024	0,350	0,667
Interaktion Jahr * Ort unter 5000 Einwohner	0,925	0,956	1,628	0,851
Interaktion Jahr * arbeitslos	0,620	0,651	0,811	0,667
*n*	*3283*	*3282*	*3282*	*3283*
*Nagelkerkes R*^*2*^	*0,036*	*0,070*	*0,050*	*0,041*

*p < 0,05, **p < 0,01, ***p < 0,001

Zu den soziodemografischen Faktoren ergeben sich folgende Haupteffekte: Ältere Befragte berichten signifikant seltener, alle Formen der partnerschaftlichen Gewalt erlebt zu haben, als jüngere Befragte. In der Westschweiz bzw. im Tessin ist im Wesentlichen die psychische Gewalt weniger verbreitet als in der deutschsprachigen Schweiz. Im ländlichen Raum, d. h. in Gemeinden mit weniger als 5000 Einwohnern, findet sich ein höheres Niveau psychischer Gewalt. Die Betroffenheit von Arbeitslosigkeit bzw. der Bezug von Arbeitslosengeld/Sozialhilfe steht zuletzt signifikant mit erhöhter psychischer Gewalt in Beziehung. Auch zu den anderen Gewaltindizes zeigen sich gewaltsteigernde Effekte dieser Variable, die allerdings nicht als signifikant ausgewiesen werden.

**Figure 1:**
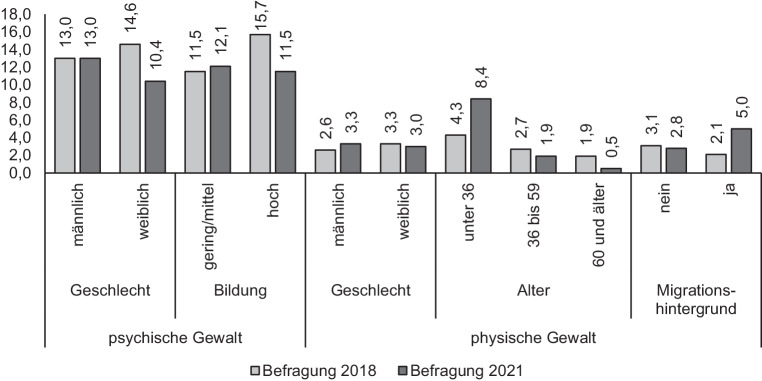

Von den insgesamt geprüften 28 Interaktionseffekten werden nur 4 als signifikant ausgewiesen. Die Entwicklungen (bzw. in diesem Fall: Nichtentwicklungen) gelten damit für die verschiedenen soziodemografischen Gruppen gleichermaßen. Gleichwohl deuten sich einige differenzielle Entwicklungen bei Betrachtung der Interaktionsvariablen an. Um diese darzustellen, finden sich in Abb. 1 Zwölfmonatsprävalenzraten für verschiedene soziodemografische Subgruppen.

Hinsichtlich der psychischen Gewalt gilt, dass bei weiblichen Befragten ein signifikanter Rückgang im Zeitvergleich eingetreten ist (von 14,6 auf 10,4 %; χ^2^ = 6,555, *p* < 0,01). Auch bei der physischen Gewalt deutet sich ein Rückgang bei weiblichen Befragten an (von 3,3 auf 3,0 %), der aber nicht signifikant ist. Bei der psychischen Gewalt findet sich laut multivariater Analyse ein signifikanter Interaktionseffekt für das Bildungsniveau. Dieser bedeutet, dass nur bei höher gebildeten Befragten ein signifikanter Rückgang von 15,7 auf 11,5 % (χ^2^ = 6,983, *p* < 0,01) vorhanden ist. Der für physische Gewalt in der multivariaten Auswertung festgestellte Interaktionseffekt für das Alter bedeutet, dass bei älteren Altersgruppen die Prävalenzrate fällt, bei jüngeren hingegen steigt: Der Anteil an unter 36-jährigen Befragten, die physische Gewalt durch den Partner oder die Partnerin erlebt haben, ist von 4,3 auf 8,4 % signifikant gestiegen (χ^2^ = 5,194, *p* < 0,05). Auch im Vergleich der Gruppen mit und ohne Migrationshintergrund deutet sich eine differenzielle Entwicklung an: Während für Befragte ohne Migrationshintergrund die Prävalenz physischer Gewaltopfererfahrungen im Zeitvergleich nahezu konstant bleibt, ist sie für Befragte mit Migrationshintergrund signifikant gestiegen (χ^2^ = 4,185, *p* < 0,05). Die Auswertungen belegen damit, dass v. a. bei jüngeren Personen die Pandemiesituation anscheinend doch mit zunehmender physischer Gewalt durch den Partner bzw. die Partnerin assoziiert ist.

## Diskussion

Anhand von 2 repräsentativen Befragungsstudien konnte erstmals für die Schweiz geprüft werden, ob die COVID-19-Pandemie einen Effekt auf das Niveau partnerschaftlicher Gewalt im Dunkelfeld hat. Die Ergebnisse entsprechen denen der Studie von Kliem et al. (2021) für Deutschland: Partnerschaftliche Gewalt hat sich im Vergleich der Befragungsjahre 2018 und 2021 nicht signifikant verändert. Für physische Gewalt und Bedrohungen finden sich nahezu konstante Zwölfmonatsprävalenzraten; psychische Gewalt ist gesunken, insbesondere bei weiblichen Befragten und Befragten mit höherer Bildung.

Zugleich bieten die vorgestellten Ergebnisse Anhaltspunkte dafür, dass die Pandemiesituation bei einigen soziodemografischen Gruppen mit erhöhter Gewalt einhergeht: Für jüngere Befragte steigt beispielsweise die Rate zur physischen Gewalt signifikant. Über die Hintergründe dieses Befundes kann nur spekuliert werden; zudem müsste er mit weiteren Studien validiert werden. Denkbar ist, dass bei jüngeren Befragten eine instabilere berufliche Situation – die jüngeren Befragten stehen eher am Anfang ihrer beruflichen Karriere – oder die häufigere Gegenwart von kleinen bzw. schulpflichtigen Kindern, die aufgrund von Schulschließungen und reduzierten Freizeitangeboten stärker auf die Familie angewiesen waren, stressauslösende bzw. stressverstärkende Momente darstellen, in deren Folge es zu Aggression und Gewalt zwischen Partner*innen gekommen sein kann.

Dass es, über die Gesamtstichproben hinweg betrachtet, keinen signifikanten Anstieg partnerschaftlicher Gewalt gegeben hat, ist vor dem Hintergrund der Einflussfaktoren dieses Verhaltens nicht völlig überraschend. Diese Einflussfaktoren liegen nur teilweise im Bereich situativer Merkmale (wie Armut, Arbeitslosigkeit, Stress und Überbelastung; Eidgenössisches Büro für die Gleichstellung von Frau und Mann 2020), – und diese situativen Merkmale haben sich für die gesamte Schweizer Gesellschaft in jüngerer Zeit auch nicht durchweg deutlich verschlechtert. So lag die Arbeitslosenquote im März 2020 beispielsweise bei 2,9 %, ein Jahr später im März 2021 nur unwesentlich höher bei 3,4 % (mit bis Oktober deutlich rückläufiger Tendenz; Staatssekretariat für Wirtschaft 2021, S. 22). Andere bedeutsame Faktoren partnerschaftlicher Gewalt wie Missbrauchs- und Gewalterfahrung in der Kindheit, eine dissoziale Persönlichkeit, Substanz- und insbesondere Alkoholkonsum, Dominanzorientierungen usw. sind hingegen Ergebnis längerfristiger, z. T. in Kindheit und Jugend zu verortender Sozialisationsprozesse (u. a. Egger und Schär Moser 2008; Eidgenössisches Büro für die Gleichstellung von Frau und Mann 2020). Die Pandemie kann sich aber nicht auf diese Sozialisationsprozesse, die in der Vergangenheit liegen, ausgewirkt haben.

Die vorgestellte Studie weist zugleich verschiedene Limitationen auf, die abschließend zu erwähnen sind. Hierzu zählt die in beiden Befragungen eher geringe Rücklaufquote. Jeweils ca. 4 von 5 zur Befragung eingeladene Personen haben sich nicht beteiligt. Es kann nicht ausgeschlossen werden, dass es sich dabei um besondere Gruppen handelt, d. h. beispielsweise besonders wenig oder besonders stark von partnerschaftlicher Gewalt betroffene Gruppen. Anhand des Anteils an Personen mit Migrationshintergrund zeigt sich ein selektiver Rücklauf: In den Stichproben liegt der Anteil bei 22,8 bzw. 16,7 %, was deutlich unter dem Bevölkerungsanteil von 37,7 % (ab 15-Jährige; Bundesamt für Statistik 2020b) liegt. Die Bevölkerung mit Migrationshintergrund wird mit den Stichproben also nur begrenzt abgebildet. Auch andere Risikogruppen für partnerschaftliche Gewalt (z. B. sozial benachteiligte Familien, Familien mit verhaltensauffälligen Kindern), bei denen sich womöglich bedeutsamere Verschlechterungen hinsichtlich der Gewalt während der zurückliegenden Monate gezeigt haben könnten, werden mit repräsentativen Befragungen vermutlich schlechter erreicht. Eine zusätzliche Limitation ist, dass aufgrund des eingesetzten Instruments, welches in binärer Weise Gewalterlebnisse erfragt, nicht geprüft werden kann, ob sich die Inzidenz bzw. Intensität partnerschaftlicher Gewalt verändert hat. Weitere differenzierte Befragungsstudien sind daher zweifellos notwendig.
